# Optimal vancomycin AUC_24_/MIC ratio for predicting clinical outcomes in patients with glycopeptide-susceptible *Enterococcus faecium* bacteremia

**DOI:** 10.1007/s10096-026-05473-w

**Published:** 2026-03-20

**Authors:** Ryo Yamaguchi, Takehito Yamamoto, Sohei Harada, Mayu Shibuya, Miyuki Mizoguchi, Yoshimi Higurashi, Yuki Miyata, Naoki Ogiue, Takeya Tsutsumi, Tappei Takada

**Affiliations:** 1 Department of Pharmacy, The University of Tokyo Hospital, 7-3-1 Hongo, Bunkyo-ku, Tokyo, 113-8655 Japan; 2 Department of Microbiology and Infectious Diseases, Toho University School of Medicine, Tokyo, Japan; 3 Department of Infection Control and Prevention, The University of Tokyo Hospital, Tokyo, Japan; 4 Department of Infectious Diseases, The University of Tokyo Hospital, Tokyo, Japan

**Keywords:** *Enterococcus faecium*, Vancomycin, AUC/MIC, Bacteremia

## Abstract

**Purpose:**

Data on the relationship between the 24-hour area under the concentration–time curve to the minimum inhibitory concentration (AUC_24_/MIC) and clinical outcomes in glycopeptide-susceptible *Enterococcus faecium* (GSEF) bacteremia remain limited. This study aimed to determine the optimal vancomycin AUC_24_/MIC threshold associated with treatment outcomes in patients with GSEF bacteremia.

**Methods:**

We retrospectively reviewed cases of GSEF bacteremia treated with vancomycin at a single tertiary care hospital between April 1, 2010, and March 31, 2024. Vancomycin AUC_24_ was estimated using a Bayesian approach, and MIC values were determined by Etest. The primary outcome was treatment failure, defined as a composite of 30-day all-cause mortality and microbiological failure (persistence of bacteremia in follow-up blood cultures).

**Results:**

A total of 67 patients were included, and treatment failure occurred in 11 (16.4%). Classification and regression tree (CART) analysis identified an AUC_24_/MIC_Etest_ threshold of ≥ 427 as a predictor for reduced treatment failure. In multivariable logistic regression, achieving AUC_24_/MIC_Etest_ ≥427 was independently associated with reduced treatment failure (odds ratio, 0.056; 95% confidence interval, 0.003–0.35; *P* = 0.01).

**Conclusion:**

These findings indicate that achieving a vancomycin AUC_24_/MIC_Etest_ ≥427 was associated with reduced treatment failure in GSEF bacteremia. Larger prospective studies are needed to validate this pharmacokinetic/pharmacodynamic target.

**Supplementary Information:**

The online version contains supplementary material available at 10.1007/s10096-026-05473-w.

## Introduction

*Enterococcus faecium* has emerged as a major cause of healthcare-associated bloodstream infections, associated with substantial morbidity and mortality; reported 30-day mortality rates range from 20% to 40% [1–3]. In the United States, attention has focused on vancomycin-resistant *E. faecium*; however, glycopeptide-susceptible strains continue to account for a considerable proportion of clinical isolates worldwide, particularly in Asia and Europe [4–7]. This global epidemiology underscores the need to optimize therapy for glycopeptide-susceptible *E. faecium* (GSEF) bacteremia to improve outcomes.

Vancomycin remains the first-line agent for GSEF bacteremia [8, 9]. Its efficacy is linked to the pharmacokinetic/pharmacodynamic (PK/PD) index defined by the ratio of the 24-hour area under the concentration–time curve to the minimum inhibitory concentration (AUC_24_/MIC) [10]. For methicillin-resistant *Staphylococcus aureus* (MRSA), recent consensus guidelines from the United States and Japan recommend targeting an AUC_24_/MIC of 400–600 to maximize efficacy while minimizing nephrotoxicity [11, 12]. Although similar PK/PD relationships have been reported in enterococcal bacteremia, available data are limited by methodological shortcomings. For example, Jumah et al. identified an optimal cutoff value of AUC_24_/MIC_Etest_ ≥389 using classification and regression tree (CART) analysis [13]. However, their cohort included both *E. faecalis* and *E. faecium*, making it uncertain whether this threshold applies specifically to GSEF bacteremia. *E. faecalis* and *E. faecium* differ in the background of affected patients and the organs involved in infection; for example, *E. faecalis* generally accounts for most cases of enterococcal infective endocarditis, whereas *E. faecium* is often encountered in healthcare-associated settings [14, 15]. Therefore, PK/PD thresholds derived from mixed-species cohorts may not be directly generalizable to GSEF bacteremia. In addition, a recent multicenter study from Japan reported that an average AUC_24_/MIC ≥ 427 independently predicted treatment success in *E. faecium* bacteremia [16]. However, in this study, all *E. faecium* isolates were reported with an MIC of 1 mg/L, while cases with MIC values ≤ 0.5 mg/L were excluded due to unclear results. Consequently, MIC variability was not taken into account, which may have led to an overestimation of drug exposure relative to the true MIC. In particular, for pathogens with lower MIC values, this limitation raises concerns about both the accuracy and the clinical applicability of the proposed PK/PD target. In one study, approximately 32% of isolates (28/87) had MICs ≤ 0.5 mg/L, indicating potential error in AUC_24_/MIC calculations within this range [17]. Collectively, these issues highlight the need for studies focusing exclusively on GSEF bacteremia that incorporate precise MIC measurements including lower concentrations. Such efforts may enable the identification of a more accurate and clinically relevant vancomycin AUC_24_/MIC target.

This study aimed to evaluate the association between vancomycin AUC_24_/MIC and treatment outcomes in patients with GSEF bacteremia and to identify an optimal PK/PD target to inform therapeutic strategies.

## Materials and methods

### Study design and setting

This was a retrospective, single-center, observational cohort study conducted at The University of Tokyo Hospital from April 1, 2010, to March 31, 2024. The study protocol was approved by the institutional ethics committee (approval No. 2023138NI), and the requirement for written informed consent was waived. The study complied with the ethical standards outlined in the 1964 Declaration of Helsinki and its subsequent amendments. Eligible patients were those with at least one positive blood culture for GSEF who received vancomycin therapy for ≥ 5 consecutive days. Patients with polymicrobial bacteremia were included. Patients were excluded if stored microbiological isolates were unavailable, there was no regrowth of *E. faecium* from the stored samples, or renal replacement therapy was received. Patients who did not receive vancomycin (i.e., treated with alternative antibiotics or no antibiotics) were not eligible for inclusion. GSEF bacteremia was defined, as in prior studies, as at least 1 blood culture positive for ampicillin-resistant, vancomycin-susceptible *E. faecium* accompanied by signs or symptoms consistent with the suspected infection site [18–20]. For patients with multiple GSEF bacteremia treatment episodes, only the first treatment episode was analyzed.

### Data collection

Demographic and clinical data were retrospectively extracted from electronic medical records. The collected variables included age, sex, height, weight, infection type (community- or hospital-acquired), ICU stay, comorbidities, Charlson Comorbidity Index (CCI), Pitt bacteremia score, and relevant laboratory data. Laboratory values included serum creatinine, serum albumin, hemoglobin, blood urea nitrogen (BUN), C-reactive protein, white blood cell count, absolute neutrophil count (ANC), and platelet count. These parameters were recorded on or immediately before the day of the first positive blood culture for *E. faecium*. Infections were defined as community-acquired if the first positive blood culture was obtained within 48 h of hospital admission; otherwise, they were categorized as hospital-acquired. The estimated glomerular filtration rate (eGFR) was calculated using the formula recommended by the Japanese Society of Nephrology [21]. Neutropenia was defined as an ANC < 0.5 × 10^3^/µL [2, 22]. Immunosuppressed status was defined as having received chemotherapy, radiation therapy, or immunosuppressive agents (e.g., calcineurin inhibitors or corticosteroids) within the 30 days preceding the positive blood culture [23]. The source of bacteremia was determined based on microbiological findings and clinical judgment, and was classified according to the CDC/NHSN surveillance definitions and criteria for specific types of infections [20]. The duration of vancomycin therapy was recorded. The implementation of source control procedures, such as endoscopic retrograde cholangiopancreatography, percutaneous transhepatic biliary drainage, or central venous catheter removal, was also documented. Source control was evaluated only in patients with conditions in which such procedures are clinically indicated, specifically cholangitis and catheter-related bloodstream infections. Appropriate antimicrobial therapy was defined as the intravenous administration of antibiotics active against all blood culture isolates, initiated within 48 h of index blood culture collection [24]. Under this definition, patients who ultimately received active therapy but started vancomycin after 48 h (e.g., after susceptibility results became available) were classified as not having received appropriate antimicrobial therapy. Co-administered antibiotics other than vancomycin were reviewed to assess the appropriateness of antimicrobial therapy.

### Microbiological data

Blood culture specimens were processed using the BACTEC FX system (Becton Dickinson Microbiology Systems, Sparks, MD, USA). Identification of *Enterococcus faecium* isolates was performed with the MALDI Biotyper 1.0 (Bruker Daltonics, Bremen, Germany). Antimicrobial susceptibility testing for ampicillin and vancomycin was conducted using the MicroScan WalkAway System (Beckman Coulter Japan, Tokyo, Japan), and results were interpreted according to Clinical and Laboratory Standards Institute (CLSI) guidelines (M100-S26) [25]. Polymicrobial bacteremia was defined as the presence of one or more additional organisms—aside from *E. faecium*—in the same blood culture specimen, provided these organisms were not deemed contaminants based on CDC criteria [20].

For this study, vancomycin MICs were determined using two methods: (1) a commercial automated broth microdilution system (MIC_WalkAway_; MicroScan WalkAway-96 Plus, Beckman Coulter), and (2) a gradient diffusion method (MIC_Etest_; Etest, bioMérieux), according to the respective manufacturer’s protocols. To ensure consistency across the study period, all stored isolates were re-tested using the MicroScan WalkAway-96 Plus system. The available MIC range in the MicroScan WalkAway-96 Plus system was limited to discrete values of ≤ 0.5, 1, 2, 4, 8, 16, and > 16 mg/L. MIC_Etest_ results were independently reviewed by three clinical microbiologists who were blinded to the clinical outcomes. If at least two reviewers agreed on the interpretation, that value was adopted. In cases where the interpretations were discrepant among all three reviewers, the isolate was retested, and the final MIC value was determined based on the same procedure applied to the repeat results. Agreement between MIC testing methods was assessed using categorical agreement and essential agreement. Categorical agreement was defined as agreement in interpretive categories (susceptible/intermediate/resistant), and essential agreement was defined as MIC values within ± 1 log_2_ dilution between methods [26]. For agreement assessment, WalkAway MIC values reported as ≤ 0.5 mg/L were treated as 0.5 mg/L.

### Vancomycin dosing and pharmacodynamic data

The hospital guidelines recommend administering vancomycin as an initial loading dose of 20–25 mg/kg, followed by maintenance dosing adjusted based on creatinine clearance (Ccr). However, the final dosing regimen was determined at the discretion of the attending physician. Pharmacist-led therapeutic drug monitoring (TDM) was routinely performed, and vancomycin Cmin was measured on or after the second day of treatment. The target Cmin range was 10–20 mg/L in accordance with a previous clinical practice guideline [27]. Serum vancomycin concentrations were measured using a particle-enhanced turbidimetric inhibition immunoassay (PETINIA) with the Dimension EXL 200 analyzer and Dimension^®^ Flex^®^ reagent cartridge VANC (Siemens Healthcare Diagnostics, Tokyo, Japan).

The area under the concentration-time curve during the first 24 h after vancomycin initiation (AUC_24_) was estimated using a Bayesian pharmacokinetic approach with the practical AUC-guided therapeutic drug monitoring (PAT) software (ver. 3.0d; Japanese Society of Chemotherapy and Japanese Society of Therapeutic Drug Monitoring, Tokyo, Japan) [28, 29]. PAT was implemented in R (version 3.6.2) and run on a Windows 10 environment, with access available on both desktop computers and smartphones. Bayesian estimation was performed using a previously published Japanese population pharmacokinetic model [30]. AUC_24_/MIC and Cmin/MIC ratios were calculated using vancomycin MIC values obtained from both the MicroScan WalkAway system and the MIC_Etest_. For isolates reported as ≤ 0.5 mg/L by the WalkAway system, MIC values were uniformly assigned as 0.5 mg/L for the purpose of PK/PD calculations.

### Clinical outcomes

The primary outcome was treatment failure, defined as a composite of (i) 30-day all-cause mortality or (ii) microbiological failure, i.e., persistent bacteremia documented by a positive follow-up blood culture obtained > 72 h after initiation of appropriate therapy in patients with at least one follow-up culture available [31, 32]. In patients without follow-up blood cultures, microbiological failure could not be assessed, and treatment failure was adjudicated solely on the basis of 30-day all-cause mortality. Patients who did not meet the failure criteria were classified as having treatment success. Secondary outcomes comprised 90-day all-cause mortality (death within 90 days of the first GSEF-positive blood culture), in-hospital mortality (death during the index admission), relapse (recurrent GSEF bacteremia within 90 days after documented microbiological clearance), and readmission within 90 days of the first GSEF-positive culture. Relapse required recurrence of GSEF bacteremia following at least one negative follow-up blood culture confirming clearance. For research purposes, all pharmacokinetic analyses (including AUC estimation) were performed retrospectively by an infectious-diseases pharmacist. MIC determinations were conducted independently by separate investigators and were linked to the clinical dataset only after outcome adjudication had been completed. Safety endpoints included: (i) acute kidney injury defined by KDIGO criteria—either a ≥ 1.5-fold increase in serum creatinine (sCr) from baseline within 7 days or an absolute increase of ≥ 0.3 mg/dL within 48 h after vancomycin initiation [33]; (ii) liver injury, defined as AST ≥ 90 U/L or ALT ≥ 126 U/L (i.e., ≥ 3× the upper limit of normal) during vancomycin therapy [34]; and (iii) other adverse events attributed to vancomycin use for GSEF bacteremia [19].

### Statistical analysis

Continuous variables were summarized as medians with interquartile ranges (IQR) and compared between the treatment-success and treatment-failure groups using the Mann–Whitney U test. Categorical variables were analyzed with Pearson’s χ² test or Fisher’s exact test, as appropriate. To identify predictors of treatment failure, we adopted a two-stage approach. First, in line with a prior vancomycin PK/PD report [13], a classification and regression tree (CART) analysis was used to determine the optimal threshold of AUC_24_/MIC_Etest_ associated with failure; this cutoff was then used to dichotomize the PK/PD index. Second, a multivariable logistic-regression model was fitted to evaluate the independent association between the dichotomized AUC_24_/MIC_Etest_ and treatment failure. To enhance clinical face validity and adjust for established prognostic factors in severe infections [35–37], the Charlson Comorbidity Index, immunosuppressed state, and Pitt bacteremia score were entered a priori (forced) as adjustment covariates. Multicollinearity was assessed using variance-inflation factors (VIFs), with VIF > 10 indicating collinearity; when collinearity was present, only one of the correlated variables was retained. Model calibration was examined with the Hosmer–Lemeshow goodness-of-fit test, and discrimination was quantified by the area under the receiver operating characteristic (ROC) curve. To assess robustness in a low-events setting and potential model instability, we conducted sensitivity analyses using (i) Firth’s bias-reduced penalized logistic regression [38] and (ii) a reduced multivariable model with fewer covariates. All analyses were conducted in R version 4.3.1 (R Foundation for Statistical Computing, Vienna, Austria) and JMP Pro 18.0.1 (SAS Institute Inc., Cary, NC, USA). Two-sided P values < 0.05 were considered statistically significant.

## Results

### Patient characteristics and clinical outcomes

**Fig. 1:**
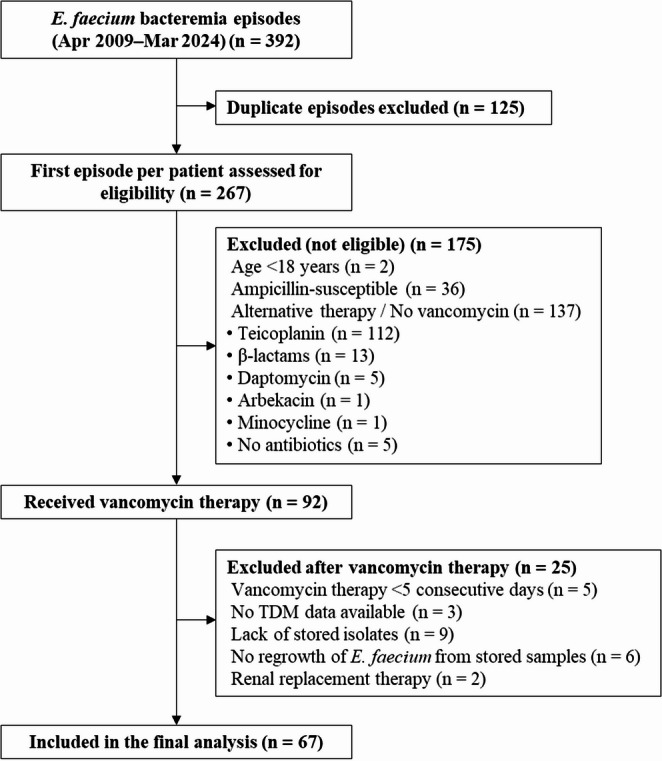
Flowchart of patient selection

A total of 67 patients with GSEF bacteremia were included in the analysis (Fig. 1). Among 267 unique first episodes assessed for eligibility, 137 were treated with alternative antibiotics or received no antibiotics and were excluded from the final cohort. In addition, five patients were excluded because vancomycin therapy was discontinued within < 5 consecutive days (early death, *n* = 1; renal dysfunction requiring a switch to an alternative agent, *n* = 2; step-down to oral linezolid, *n* = 2).

The baseline characteristics of the cohort are presented in Table 1. The median age was 68.0 years (IQR, 52.0–75.0), and 70.1% were male. There were no statistically significant differences between the success and failure groups in most baseline demographics or comorbidities. However, the failure group had significantly higher neutrophil counts (9.5 vs. 4.7 × 10³/mm³; *P* = 0.048) and a shorter length of hospital stay (22.0 vs. 43.0 days; *P* = 0.024). The most common sources of bacteremia were hepatobiliary (47.8%) and febrile neutropenia without obvious source (22.4%). No cases of central nervous system infection or infective endocarditis were documented in the study cohort. Polymicrobial bacteremia was observed in 25.4% of patients. Appropriate antimicrobial therapy was administered in 82.1% of cases. The remaining patients were classified as not receiving appropriate therapy because vancomycin was initiated more than 48 h after index blood culture collection, typically after susceptibility results became available. Source control was achieved in 81.6%. The primary outcome, treatment failure, was observed in 11 of 67 patients (16.4%). Follow-up blood cultures were performed in 60 of 67 cases (90%). The overall 30-day mortality rate was 13.4% (9/67).

**Table 1:** Baseline characteristics of patients

Characteristic	All (*n* = 67)	Success (*n* = 56)	Failure (*n* = 11)	*P* value
Male sex	47 (70.1)	40 (71.4)	7 (63.6)	0.606
Age (years)	68.0 (52.0–75.0)	68.0 (52.0–77.0)	63.0 (47.0–72.0)	0.290
Height (cm)	163.9 (156.8–170.0)	163.7 (156.9–168.3)	164.0 (154.5–173.2)	0.515
Weight (kg)	53.7 (47.0–62.7)	53.9 (47.3–62.5)	52.1 (46.3–63.8)	0.926
ICU stay	9 (13.4)	7 (12.5)	2 (18.2)	0.613
Mechanical ventilation	5 (7.5)	3 (5.4)	2 (18.2)	0.139
Hospital-acquired infection^a^	54 (80.6)	47 (83.9)	7 (63.6)	0.120
Charlson Comorbidity Index	3.0 (2.0–6.0)	3.0 (2.0–5.0)	3.0 (3.0–8.0)	0.159
Pitt bacteremia score	1.0 (0.0–2.0)	1.0 (0.0–2.0)	2.0 (0.0–5.0)	0.477
Hypertension	21 (31.3)	17 (30.4)	4 (36.4)	0.695
Chemotherapy	27 (40.3)	22 (39.3)	5 (45.5)	0.703
COPD	3 (4.5)	3 (5.4)	0 (0.0)	0.432
WBC count (×10^3^/µL)	6.5 (1.5–11.6)	6.2 (1.1–10.3)	11.6 (5.0–14.4)	0.076
Neutrophil count (×10^3^/µL)	4.8 (1.1–9.5)	4.7 (0.8–8.6)	9.5 (3.8–12.5)	0.048
Neutropenia^b^	14 (20.9)	13 (23.2)	1 (9.1)	0.292
Hemoglobin (g/dL)	8.9 (7.7–10.3)	8.9 (7.7–10.2)	9.3 (7.9–11.3)	0.504
Platelet count (×10^4^/µL)	15.6 (3.6–23.5)	15.6 (3.3–22.7)	15.6 (8.7–30.3)	0.352
BUN (mg/dL)	13.8 (10.2–19.4)	13.7 (9.9–18.1)	13.9 (10.2–29.5)	0.472
Serum creatinine (mg/dL)	0.7 (0.5–0.9)	0.7 (0.5–0.8)	0.6 (0.4–0.9)	0.461
eGFR (mL/min/1.73 m^2^)	85.0 (61.1–109.3)	84.3 (61.8–101.9)	104.7 (58.3–133.5)	0.348
Serum albumin level (g/dL)	2.4 (2.1–3.0)	2.5 (2.1–3.0)	2.3 (1.9–2.8)	0.274
CRP (mg/dL)	6.7 (3.6–11.7)	6.9 (3.9–11.4)	6.2 (2.4–14.6)	0.780
Immunosuppressed state^c^	33 (49.3)	28 (50.0)	5 (45.5)	0.783
Source of infection				
Hepatobiliary	32 (47.8)	23 (41.1)	9 (81.8)	0.245
Intravascular catheter	6 (9.0)	6 (10.7)	0 (0.0)	
Febrile neutropenia	15 (22.4)	14 (25.0)	1 (9.1)	
Intra-abdominal	8 (11.9)	7 (12.5)	1 (9.1)	
Surgical site	3 (4.5)	3 (5.4)	0 (0.0)	
Others^d^	3 (4.5)	3 (5.4)	0 (0.0)	
Polymicrobial infections	17 (25.4)	14 (25.0)	3 (27.3)	0.874
Appropriateness of therapy	55 (82.1)	45 (80.4)	10 (90.9)	0.404
Duration of therapy (days)	15.0 (11.0–18.0)	14.5 (12.0–21.0)	15.0 (9.0–17.0)	0.564
Source control, n/N_eligible (%)^e^	31/38 (81.6)	25/29 (86.2)	6/9 (66.7)	0.186
Length of hospital stay (days)	42.0 (24.0–85.0)	43.0 (26.5–87.0)	22.0 (20.0–44.0)	0.024

Data are presented as medians (IQR) for continuous variables and *n* (%) for categorical variables unless otherwise stated

^a^Hospital-acquired infection was defined as a positive blood culture obtained more than 48 h after hospital admission

^b^Neutropenia was defined as a neutrophil count of < 500/mm^3^

^c^Use of chemotherapy and/or immunosuppressive drugs (corticosteroids–cyclosporin A/tacrolimus) within 1 month before bacteremia

^d^Urinary tract infection (1); Unclear (2)

^e^Source control was assessed only in patients eligible for source control (e.g., endoscopic retrograde cholangiopancreatography [ERCP], percutaneous transhepatic biliary drainage [PTBD], or central venous catheter removal)

Abbreviations: *BUN* blood urea nitrogen, *COPD* chronic obstructive pulmonary disease, *WBC* white blood cell, *eGFR* estimated glomerular filtration rate, *CRP* C-reactive protein

### PK/PD analysis and target identification

**Fig. 2:**
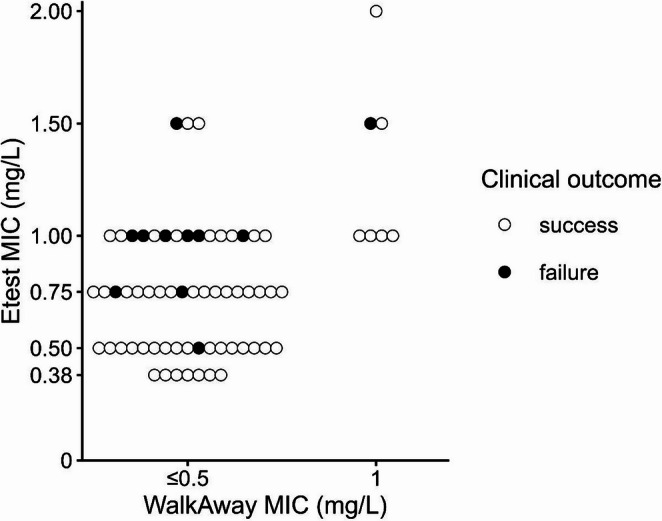
Comparison of Vancomycin MIC values between Etest and WalkAway according to clinical outcomes. Scatter plot of vancomycin MIC values determined by Etest and the MicroScan WalkAway system. Each circle represents an individual isolate; black circles indicate treatment failure, and white circles indicate treatment success. Etest MIC values are plotted on the y-axis, and corresponding WalkAway results (≤0.5 or 1 mg/L) are plotted on the x-axis. In accordance with the study protocol, two isolates underwent repeat Etest measurements, and all retest results were within a two-fold range of the initial values.

Figure 2 illustrates the comparison of vancomycin MIC values between Etest and WalkAway according to clinical outcomes. Etest-derived MIC values were significantly higher in the treatment failure group compared with the success group (1.00 [IQR, 0.88–1.00] vs. 0.75 [IQR, 0.50–1.00] mg/L; *P* = 0.009). In contrast, WalkAway MIC results were largely clustered at ≤0.5 mg/L in both groups (91% in failure and 89% in success), with only a few isolates reported as 1.0 mg/L. Overall, vancomycin MICs determined by Etest tended to be systematically higher than those obtained by WalkAway. Categorical agreement for vancomycin susceptibility was 100% between WalkAway and Etest, whereas essential agreement was 95.5% (64/67 within ± 1 log_2_ dilution; Table S1). Since the majority of MIC_WalkAway_ (approximately 90%) were ≤ 0.5 mg/L, subsequent PK/PD analyses were conducted using Etest-derived MIC values.

**Fig. 3:**
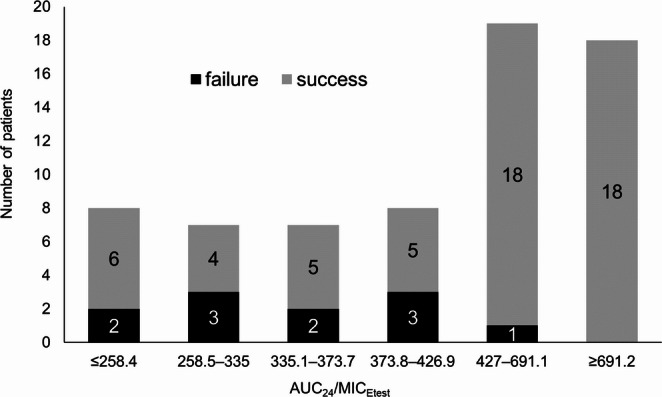
Distribution of clinical outcomes according to AUC_24_/MIC_Etest_ values. Stacked bar graph showing the number of patients with treatment success (gray) and treatment failure (black), stratified by AUC_24_/MIC_Etest_ ranges. Abbreviations: AUC, area under the curve

We next compared vancomycin PK/PD indices between groups (Table 2). Among the 67 patients, the median AUC_24_/MIC_Etest_ was significantly higher in the success group than in the failure group (537.5 [IQR, 367.5–719.8] vs. 357.5 [IQR, 278.0–397.2]; *P* = 0.004). No significant differences were observed in AUC_24_ values alone or AUC_24_/MIC_WalkAway_. Similarly, although trough concentrations (Cmin) alone or Cmin/MIC_WalkAway_ were not significantly different between success and failure groups, Cmin/MIC_Etest_ was significantly higher in the success group (23.7 [IQR, 12.9–27.7] vs. 11.7 [IQR, 9.0–17.3]; *P* = 0.013). Based on these results, CART analysis was performed to identify the optimal AUC_24_/MIC_Etest_ cutoff for predicting treatment failure, which was determined to be 427. Figure 3 depicts the distribution of clinical outcomes across different AUC_24_/MIC_Etest_ ranges, demonstrating a marked reduction in treatment failure among patients with an AUC_24_/MIC_Etest_ ≥ 427.

**Table 2:** Vancomycin PK/PD parameters

PK/PD parameters	Success (*n* = 56)	Failure (*n* = 11)	*P* value
MIC_Etest_	0.75 (0.50–1.00)	1.00 (0.88–1.00)	0.009
MIC_WalkAway_ ≤0.5 mg/L	50 (89.3)	10 (90.9)	1.0
Cmin (mg/L)	13.8 (10.4–18.2)	11.7 (9.3–17.2)	0.576
Cmin/MIC_WalkAway_	25.1 (19.2–32.4)	23.4 (14.4–34.4)	0.748
Cmin/MIC_Etest_	23.7 (12.9–27.7)	11.7 (9.0–17.3)	0.013
AUC_24_ (mg·h/L)	357.0 (319.2–405.0)	324.9 (315.4–374.4)	0.352
AUC_24_/MIC_WalkAway_	694.4 (542.0–798.3)	649.7 (567.9–748.7)	0.542
AUC_24_/MIC_Etest_	537.5 (367.5–719.8)	357.5 (278.0–397.2)	0.004
AUC_24_/MIC_Etest_ ≥427	36 (64.3)	1 (9.1)	0.002

Data are presented as medians (IQR) for continuous variables and *n* (%) for categorical variables unless otherwise stated

Abbreviations: *PK* pharmacokinetics, *PD* pharmacodynamics, *MIC* minimum inhibitory concentration, *Cmin* minimum trough concentration, *AUC* area under the curve

### Independent predictors of treatment failure

The results of the multivariable logistic regression analysis are shown in Table 3. After adjustment for the Charlson Comorbidity Index, immunosuppressed state, and Pitt bacteremia score, achieving an AUC_24_/MIC_Etest_ ≥427 remained independently associated with reduced treatment failure (OR, 0.056; 95% CI, 0.003–0.35; *P* = 0.01). In this model, none of the other covariates were significantly associated with the outcome. All variables included in the final model had VIFs below 2, indicating no evidence of multicollinearity. Model calibration was assessed using the Hosmer–Lemeshow goodness-of-fit test, which demonstrated good fit (*P* = 0.2). Discrimination, evaluated by the area under the receiver operating characteristic curve, was 0.833, indicating good model performance. Sensitivity analyses using Firth’s penalized logistic regression and a reduced multivariable model excluding immunosuppressed state yielded directionally consistent results, and AUC_24_/MIC_Etest_ ≥427 remained significantly associated with reduced treatment failure (Tables S2 and S3).

**Table 3:** Logistic regression analysis of risk factors associated with vancomycin treatment failure

Variables	Univariable		Multivariable	
	Odds ratio (95% CI)	*P* value	Odds ratio (95% CI)	*P* value
Pitt bacteremia score	1.31 (0.98–1.76)	0.061	1.26 (0.90–1.81)	0.181
Charlson Comorbidity Index	1.17 (0.92–1.50)	0.189	1.23 (0.90–1.77)	0.209
Immunosuppressed state	0.83 (0.22–3.08)	0.783	2.60 (0.48–17.80)	0.288
AUC_24_/MIC_Etest_≥427	0.056 (0.003–0.32)	0.008	0.056 (0.003–0.35)	0.010

Abbreviations: *CI* confidence interval, *MIC* minimum inhibitory concentration, *AUC* area under the curve

### Clinical outcomes according to the PK/PD target

To assess the clinical significance of this PK/PD target, patients were stratified by the cutoff value, and clinical outcomes were compared (Table 4). Patients who achieved the target (AUC_24_/MIC_Etest_ ≥427) had a significantly lower incidence of treatment failure than those who did not (2.7% vs. 33.3%; *P* = 0.002). To examine the relationship between drug exposure and toxicity, AUC_24_ values were compared according to the presence or absence of adverse events. Acute kidney injury occurred in 13.4% of patients (9/67), and hepatotoxicity occurred in 3.0% (2/67). No significant difference in the incidence of nephrotoxicity was observed between patients with AUC_24_/MIC_Etest_ ≥427 and those with AUC_24_/MIC_Etest_ <427 (10.8% vs. 16.7%; *P* = 0.485). Median AUC_24_ values did not differ significantly between patients with nephrotoxicity and those without (374.2 vs. 354.6 mg·h/L; *P* = 0.79). Similarly, no significant associations were observed between drug exposure and the incidence of hepatotoxicity or other drug-related adverse events.

**Table 4:** Clinical outcomes according to PK/PD exposure

Outcomes	AUC_24_/MIC_Etest_ <427 (*n* = 30)	AUC_24_/MIC_Etest_ ≥427 (*n* = 37)	*P* value
Treatment failure	10 (33.3)	1 (2.7)	0.002
30-day mortality	8 (26.7)	1 (2.7)	0.008
90-day mortality	11 (36.7)	8 (21.6)	0.277
In-hospital mortality	6 (20.0)	4 (10.8)	0.324
Relapse	2 (6.7)	3 (8.1)	1.0
Readmission within 90 days after bacteremia	7 (23.3)	9 (24.3)	1.0

Data are presented as *n* (%) for categorical variables

Abbreviations: *AUC* area under the curve, *MIC* minimum inhibitory concentration

## Discussion

This study investigated the relationship between vancomycin PK/PD parameters and clinical outcomes in patients with GSEF bacteremia. We utilized Etest to measure MIC values and found that an AUC_24_/MIC_Etest_ ≥427 was associated with reduced treatment failure and remained an independent predictor in multivariable analysis. Our findings may contribute to the refinement of vancomycin PK/PD optimization strategies in GSEF bacteremia.

The 30-day mortality in our cohort (13.4%) was lower than the 20–40% reported in several prior studies [1–3]. This discrepancy may be partly explained by differences in patient characteristics and clinical context: nearly half of our cases were hepatobiliary infections (47.8%), the median Pitt bacteremia score was low (1.0 [IQR, 0.0–2.0]), and source control was achieved in a high proportion of patients (81.6%), all of which may have contributed to favorable outcomes [37, 39]. In addition, although our inclusion criteria required ≥ 5 days of vancomycin therapy and excluded patients receiving renal replacement therapy, only five patients were excluded due to < 5 days of therapy (including one early death) and two were excluded due to renal replacement therapy; therefore, selection bias due to exclusion of early deaths or severe cases is likely limited.

The accuracy and resolution of MIC measurements played a critical role in the predictive performance of PK/PD indices in this study. As shown in Fig. 2, vancomycin MIC values determined using the MicroScan WalkAway system were mostly observed at ≤ 0.5 mg/L, offering limited resolution. In contrast, Etest-based measurements provided a broader distribution, ranging from 0.38 to 2.0 mg/L. This expanded range allowed for more precise calculation of AUC_24_/MIC and facilitated improved stratification of clinical outcomes. Indeed, MIC_Etest_, Cmin/MIC_Etest_, and AUC_24_/MIC_Etest_ were all significantly different between the success and failure groups, whereas Cmin/MIC_WalkAway_, AUC_24_/MIC_WalkAway_, raw Cmin, and AUC_24_ values alone were not (Table 2). While Etest provides higher resolution MIC values, it is not routinely used in many clinical laboratories, where automated systems such as WalkAway predominate. These systems report MIC values below the detection threshold as “below the threshold,” offering limited resolution for PK/PD-guided dosing. In such settings, targeting an AUC of approximately 400, as recommended for MRSA infections, may be a pragmatic alternative. Previous studies have shown that Etest tends to yield systematically higher MIC values than broth microdilution for both MRSA and *Enterococcus* species, which is consistent with our findings [40, 41]. Therefore, in regions where low MICs are common, Etest-based MIC assessment may enhance prognostic discrimination and support individualized dosing strategies that potentially reduce nephrotoxicity risk. In this study, the median MIC_Etest_ was 0.75 mg/L; thus, whereas an upper limit of 600 mg·h/L is recommended for MRSA to mitigate nephrotoxicity, maintaining an AUC of approximately 400 mg·h/L would still be sufficient to exceed the AUC_24_/MIC_Etest_ threshold of 427 when MICs are low. This finding suggests that effective therapy may be achieved at lower AUC levels while simultaneously minimizing the risk of nephrotoxicity.

In the United States, clinical guidelines for MRSA recommend AUC-guided dosing as the preferred approach for vancomycin therapy, aiming to optimize efficacy while minimizing nephrotoxicity [11]. However, the implementation of full AUC-based monitoring remains limited in many institutions because multiple sampling is difficult in routine practice due to cost and the burden on both patients and healthcare providers [42, 43]. Consequently, Cmin has remained a widely used surrogate marker in clinical practice. In this study, while absolute Cmin values did not differ significantly between the success and failure groups, the Cmin/MIC_Etest_ ratio was significantly associated with treatment success. This underscores the importance of interpreting Cmin in relation to MIC values to approximate pharmacodynamic exposure. In settings where AUC estimation is not feasible, using Cmin/MIC may offer a practical alternative. Given that the median MIC_Etest_ in our cohort was 0.75 mg/L, targeting a Cmin of 10–15 mg/L would be sufficient to achieve the AUC_24_/MIC threshold of 427. Based on our cohort data (Table 2), patients with Cmin values around 12–14 mg/L had corresponding AUC_24_ values of approximately 330–360 mg·h/L. When divided by the median MIC_Etest_ of 0.75 mg/L, these values yield an AUC_24_/MIC_Etest_ ratio of 440–480, exceeding the threshold of 427. This supports the rationale that targeting a Cmin of 10–15 mg/L would generally ensure attainment of the PK/PD target. This range aligns with conventional therapeutic trough levels and may also help minimize nephrotoxicity. These findings support the utility of Cmin-based PK/PD optimization strategies, particularly in resource-constrained settings or institutions lacking access to AUC-guided monitoring infrastructure.

Regarding safety outcomes, nephrotoxicity occurred in a subset of patients, but its incidence was not significantly associated with attainment of the AUC_24_/MIC_Etest_ ≥427 target or with overall drug exposure. Moreover, median AUC_24_ values did not differ significantly between patients with and without nephrotoxicity, and no clear associations were found between overall drug exposure and the occurrence of hepatotoxicity or other adverse events. These findings suggest that optimization of vancomycin dosing to achieve the proposed pharmacodynamic target is unlikely to increase the risk of treatment-related toxicity.

Although this study has important clinical implications, several limitations must be considered. First, as a retrospective study, residual confounding by unmeasured factors cannot be excluded despite adjustment for potential confounders in the multivariable analysis. Second, microbiological failure could not be assessed in all cases, which may have led to misclassification of the primary outcome; however, this applied to only 7 patients (10%), suggesting that the overall impact on our results was likely limited. Third, this was a single-center study conducted at a university hospital, where nearly half of the patients were immunocompromised, limiting the generalizability of the findings. Moreover, no cases of central nervous system infection or infective endocarditis were included in our cohort. Because vancomycin exposure targets are often set higher for serious infections or difficult-to-penetrate infection sites such as meningitis and endocarditis in other clinical settings [44, 45], our proposed PK/PD threshold may not be directly extrapolated to these infections. Fourth, the sample size was relatively small, with only 11 treatment failure events, which may have limited our ability to identify additional significant predictors and increased the risk of overfitting and unstable coefficient estimates, as suggested by the wide confidence intervals for some covariates. To address this concern, we conducted sensitivity analyses using Firth’s bias-reduced penalized logistic regression and a reduced multivariable model, which supported the robustness of the association between AUC_24_/MIC_Etest_ ≥427 and reduced treatment failure. Nevertheless, our findings should be interpreted as exploratory and require validation in larger cohorts. Fifth, AUC_24_ was estimated using a Bayesian approach with a single trough concentration, which is less precise than multi-sample methods. Additionally, because we used only the first available trough concentration and did not systematically incorporate subsequent measurements, we could not evaluate time-varying exposure after dose adjustments during therapy. As vancomycin TDM is typically repeated after dosage adjustments, our PK/PD indices may not fully reflect exposure throughout the entire treatment course. While practical in clinical settings, this limitation warrants caution in interpreting our PK/PD findings, and prospective studies with multi-sample AUCs are needed for validation. Despite these limitations, this study overcomes some of the evidence gap of previous studies by exploring vancomycin AUC_24_/MIC_Etest_ in GSEF bacteremia. The findings provide evidence supporting the importance of PK/PD-based regimen design in the treatment of GSEF bacteremia.

In conclusion, this study demonstrated that achieving a vancomycin AUC_24_/MIC_Etest_ ≥427 was associated with reduced treatment failure in patients with GSEF bacteremia without increasing adverse events. Larger prospective studies are warranted to validate this PK/PD target and optimize both clinical outcomes and safety.

## Supplementary Information

Below is the link to the electronic supplementary material.

Supplementary Material 1

## Data Availability

The datasets generated and/or analyzed during the current study are not publicly available due to institutional and ethical restrictions but are available from the corresponding author on reasonable request.

## Declarations

### Generative AI and AI-assisted technologies in the manuscript preparation process

During the preparation of this work the authors used ChatGPT 5 (OpenAI) and Gemini 2.5 Pro (Google) in order to polish the English language and improve readability. After using these tools, the authors reviewed and edited the content as needed and take full responsibility for the content of the published article.
