# Supplementary material for: Optimal vancomycin AUC24/MIC ratio for predicting clinical outcomes in patients with glycopeptide-susceptible Enterococcus faecium bacteremia
Source: Eur J Clin Microbiol Infect Dis. 2026 Mar 20;45(7):1929–39. doi: 10.1007/s10096-026-05473-w (PMC13328135; doi:10.1007/s10096-026-05473-w)
Supplement: Supplementary file 1 — Supplementary Material 1 [file 10096_2026_5473_MOESM1_ESM.pdf]

Supplementary Information

Optimal Vancomycin AUC_{24}/MIC Ratio for Predicting Clinical Outcomes in Patients with Glycopeptide-Susceptible *Enterococcus faecium* Bacteremia

European Journal of Clinical Microbiology & Infectious Diseases

Ryo Yamaguchi, Takehito Yamamoto, Sohei Harada, Mayu Shibuya, Miyuki Mizoguchi, Yoshimi Higurashi, Yuki Miyata, Naoki Ogiue, Takeya Tsutsumi, Tappei Takada

Affiliations:

Department of Pharmacy, The University of Tokyo Hospital, Tokyo, Japan

Department of Microbiology and Infectious Diseases, Toho University School of Medicine, Tokyo, Japan

Department of Infection Control and Prevention, The University of Tokyo Hospital, Tokyo, Japan

Department of Infectious Diseases, The University of Tokyo Hospital, Tokyo, Japan

Corresponding author: Ryo Yamaguchi, ryamaguchi-tky@g.ecc.u-tokyo.ac.jp

Table S1. Comparison of vancomycin MIC values determined by MicroScan WalkAway and Etest (n = 67).

Etest MIC (mg/L)	WalkAway MIC ≤0.5, n	WalkAway MIC 1, n	Total, n
0.38	7	0	7
0.5	17	0	17
0.75	18	0	18
1.0	15	4	19
1.5	3	2	5
2.0	0	1	1
Total	60	7	67

Data are presented as number of isolates (n). Categorical agreement was defined as agreement in interpretive categories (susceptible/intermediate/resistant), and essential agreement as MIC values within ±1 log_2 dilution between methods. For agreement assessment, WalkAway MIC values reported as ≤0.5 mg/L were treated as 0.5 mg/L. Categorical agreement was 100% (all isolates were vancomycin susceptible by both methods), and essential agreement was 95.5% (64/67).

Abbreviations: MIC, minimum inhibitory concentration.

Table S2. Sensitivity analysis using Firth's penalized logistic regression.

Variables	Univariable		Multivariable	
	**Odds ratio (95% CI)**	***P* value**	**Odds ratio (95% CI)**	***P* value**
Pitt bacteremia score	1.29 (0.99–1.71)	0.057	1.22 (0.91–1.71)	0.185
Charlson Comorbidity Index	1.17 (0.93–1.48)	0.184	1.20 (0.90–1.66)	0.229
Immunosuppressed state	0.85 (0.23–2.97)	0.793	2.21 (0.47–12.69)	0.323
$AUC_{24}/MIC_{Etest} \geq 427$	0.08 (0.008–0.38)	<0.001	0.095 (0.01–0.46)	0.002

Abbreviations: CI, confidence interval; AUC, area under the concentration–time curve; MIC, minimum inhibitory concentration.

Table S3. Sensitivity analysis using a reduced multivariable logistic regression model excluding immunosuppressed state.

Variables	Univariable		Multivariable	
	Odds ratio (95% CI)	*P* value	Odds ratio (95% CI)	*P* value
Pitt bacteremia score	1.31 (0.98–1.76)	0.061	1.21 (0.88–1.71)	0.243
Charlson Comorbidity Index	1.17 (0.92–1.50)	0.189	1.15 (0.87–1.54)	0.332
$AUC_{24}/MIC_{Etest} \geq 427$	0.056 (0.003–0.32)	0.008	0.064 (0.003–0.39)	0.012

Abbreviations: CI, confidence interval; AUC, area under the concentration–time curve; MIC, minimum inhibitory concentration.
